# A Case of a Rheumatoid Arthritis Patient on Newly Initiated Tumor Necrosis Factor Inhibitor Treatment Developing Granulomatosis With Polyangiitis Vasculitis: A Conundrum About Disease Overlap or Drug-Induced Vasculitis

**DOI:** 10.7759/cureus.72277

**Published:** 2024-10-24

**Authors:** Jessica Daza, Joud Enabi, Cristine Arcilla, Myint Thway, Gurjit S Kaeley

**Affiliations:** 1 Internal Medicine, University of Texas Rio Grande Valley School of Medicine, Weslaco, USA; 2 Internal Medicine, Texas Tech University Health Sciences Center, Odessa, USA; 3 Internal Medicine/Rheumatology, University of Florida College of Medicine – Jacksonville, Florida, USA

**Keywords:** granulomatosis with polyangitis, granulomatosis with rheumatoid arthritis, rare cases coexistence rheuamtoid arthritis with granulomatosis with polyangitis, vasculitis, vasculitis induced tumor necrosis factor-inhibitors

## Abstract

Granulomatosis with polyangiitis (GPA) is a necrotizing vasculitides subset of antineutrophil cytoplasmic antibody (ANCA)-associated vasculitis (AAVs) that involves small-sized arteries affecting multisystemic organs. Rheumatoid arthritis (RA) is a chronic autoimmune disease characterized by inflammatory polyarthritis involving the small joints. GPA and RA can have overlapping clinical presentations, including vasculitis, ocular inflammation, interstitial lung disease, and arthritis, but existing evidence indicates they are distinct conditions. Vasculitis-induced biologic tumor necrosis factor (TNF) inhibitors have been reported, particularly cutaneous vasculitis and lupus-like syndromes. We retrospectively review a case of a patient with rheumatoid arthritis with a newly diagnosed GPA with RA on anti-TNF therapy. The coexistence of RA with GPA is rare, and cases of TNF inhibitors have been reported; it becomes a conundrum when a patient faces this presentation.

## Introduction

Granulomatosis with polyangiitis (GPA) is a necrotizing vasculitides subset of antineutrophil cytoplasmic antibody (ANCA) associated vasculitis (AAVs) involving small-sized arteries, among which the most common and severely affected organs include the upper and lower respiratory tract and the kidneys. The prevalence of granulomatosis with polyangiitis (GPA) ranges from 2.3 to 146.0 cases per million persons [1]. Rheumatoid arthritis (RA) is a chronic autoimmune disease characterized by an inflammatory symmetric polyarthritis affecting the small joints with extraarticular manifestations. GPA and RA may have similar clinical presentations, including cutaneous vasculitis, ocular inflammation, and arthritis, but existing evidence indicates they are distinct conditions. The coexistence of GPA and RA is rare [2]. Vasculitis can occur denovo or related to cases with concurrent therapy such as tumor necrosis factor (TNF)-inhibitors [3].

Another condition associated with small to medial vessel vasculitis is rheumatoid vasculitis (RV), a rare, destructive inflammatory process at the blood vessel's center. Clinical reports have estimated the prevalence of RA vasculitis to be less than 1% to 5% [4]. It involves medium and small vessels, leading to necrosis. Clinical features are nail fold capillary infarcts, and it can lead to digital gangrene; classic skin lesions of RV are deep cutaneous ulcers on the lower extremities [5]. This entity can be differentiated from GPA because palpable purpura alongside lung nodules are not part of its clinical manifestations, and longstanding severe RA needs to be present. These two diseases can have similar autoimmune markers present. However, the diagnosis is based on clinical correlations such as a longstanding RA with new onset or worsening constitutional symptoms, and the finding of cutaneous ulcers, nail fold ischemia, or systemic vasculitis strongly suggests RV. 

GPA should be suspected in patients with constitutional symptoms and clinical evidence of glomerulonephritis, purpura, upper or lower respiratory tract involvement, or multiple mononeuropathy. The approach to initial therapy depends mainly upon the severity of the disease and the severity of the involved organ systems. Immunosuppressive treatment is warranted in almost all patients with active GPA.

## Case presentation

This is the case of a 33-year-old female with a seven-year history of seropositive rheumatoid arthritis with extra-articular manifestations of diffuse anterior scleritis and bilateral anterior uveitis. She was previously on prednisone 10 mg daily and methotrexate 15 mg every week plus daily folic acid for only six months and was eventually discontinued due to intolerance. She was only taking over-the-counter pain medications.

**Figure 1:**
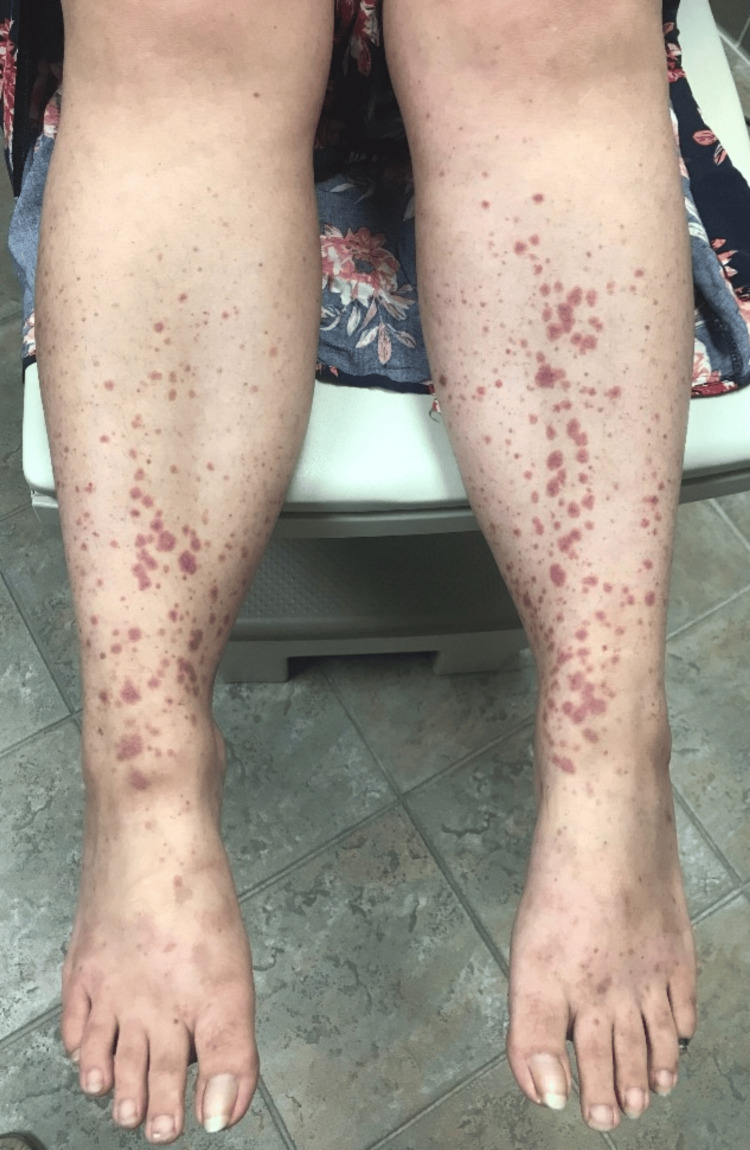
Lower extremities palpable purpura. Picture authorized by the patient.

**Figure 2:**
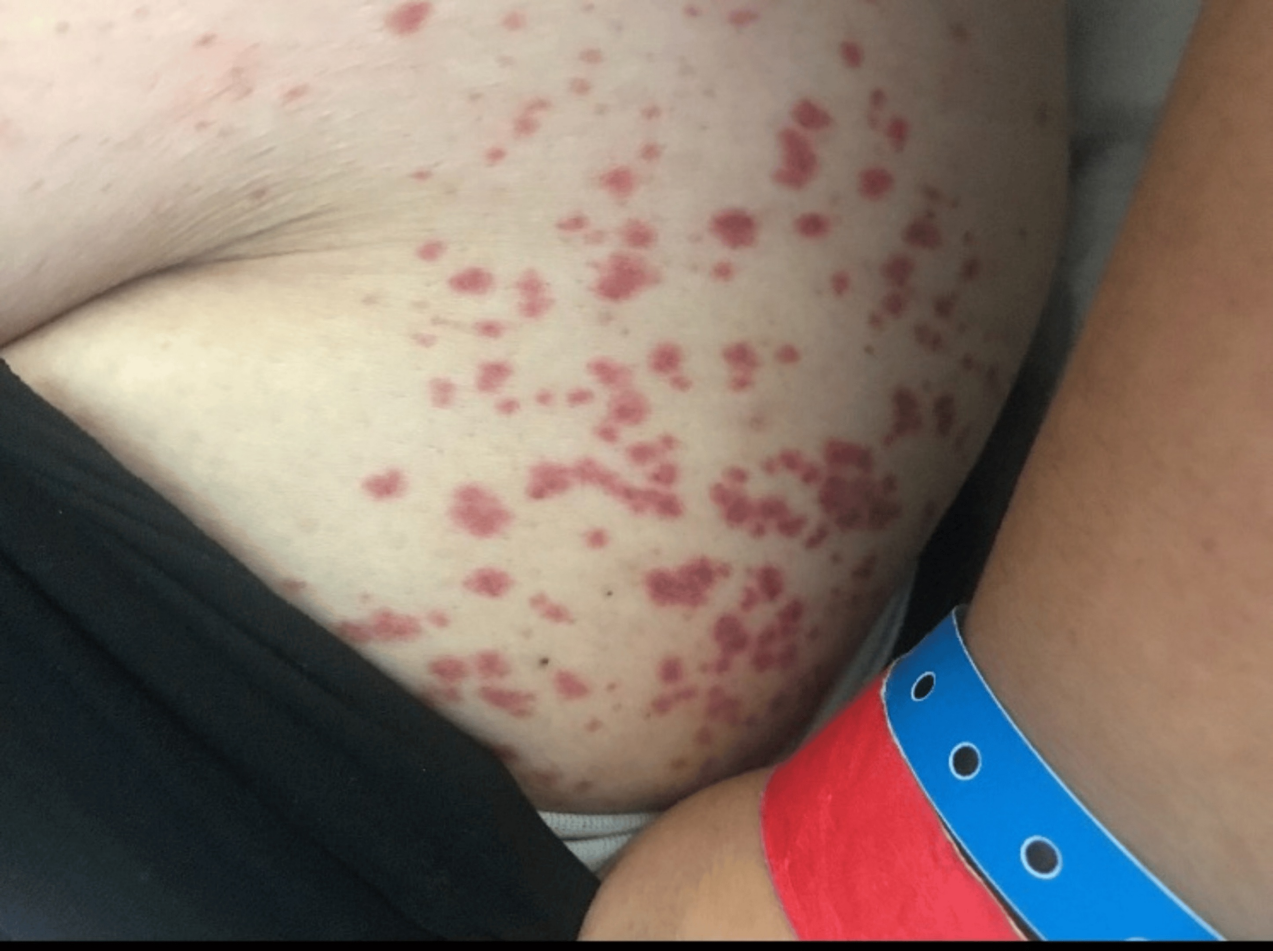
Left gluteal region palpable purpura. Picture authorized by the patient.

**Figure 3:**
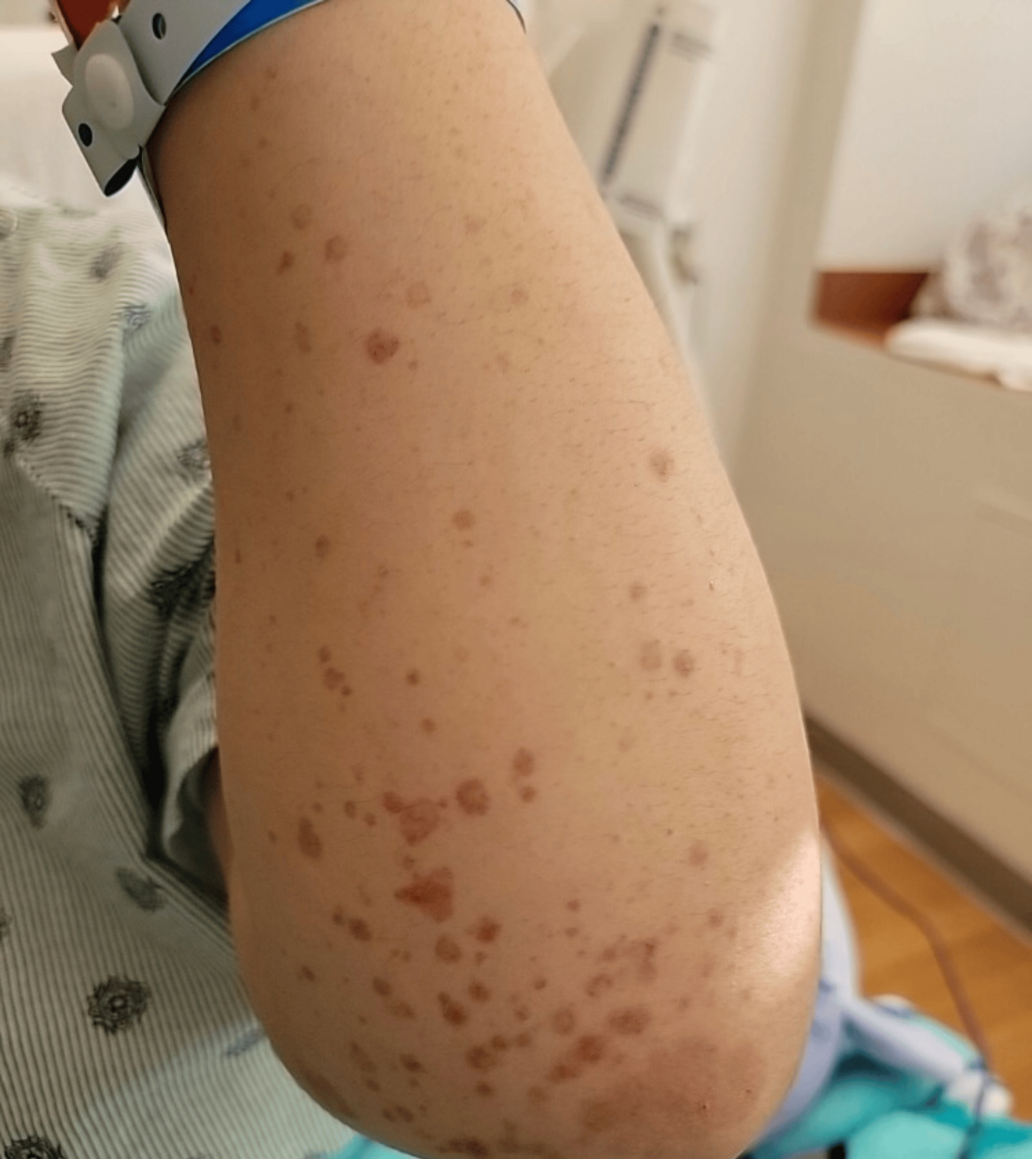
Left forearm palpable purpura. Picture authorized by the patient.

A week before admission, she was switched to an insurance-mandated adalimumab biosimilar and received one dose. She developed palpable purpura, initially on the feet and ankles, that spread to her legs and thighs (Figures 1-3), along with unintentional weight loss of more than 10 pounds. She also complained of diffuse joint pain and swelling, primarily on her hands, wrists, ankles, and feet. She denied fever, chills, sinus drainage, chest pain, dyspnea, hemoptysis, gross hematuria, hematochezia, muscle weakness, or melena. Upon admission, examination showed hypertension of 169/103 mmHg, tachycardia of 114 bpm, respiratory rate of 18 resp/min, and afebrile (97.3 F) with normal oxygen saturation. Lung auscultation was notable, with mildly diminished breath sounds at both bases. Skin had a diffuse, non-blanching palpable purpura involving bilateral lower and upper extremities, lower back, and flank areas. 

**Figure 4:**
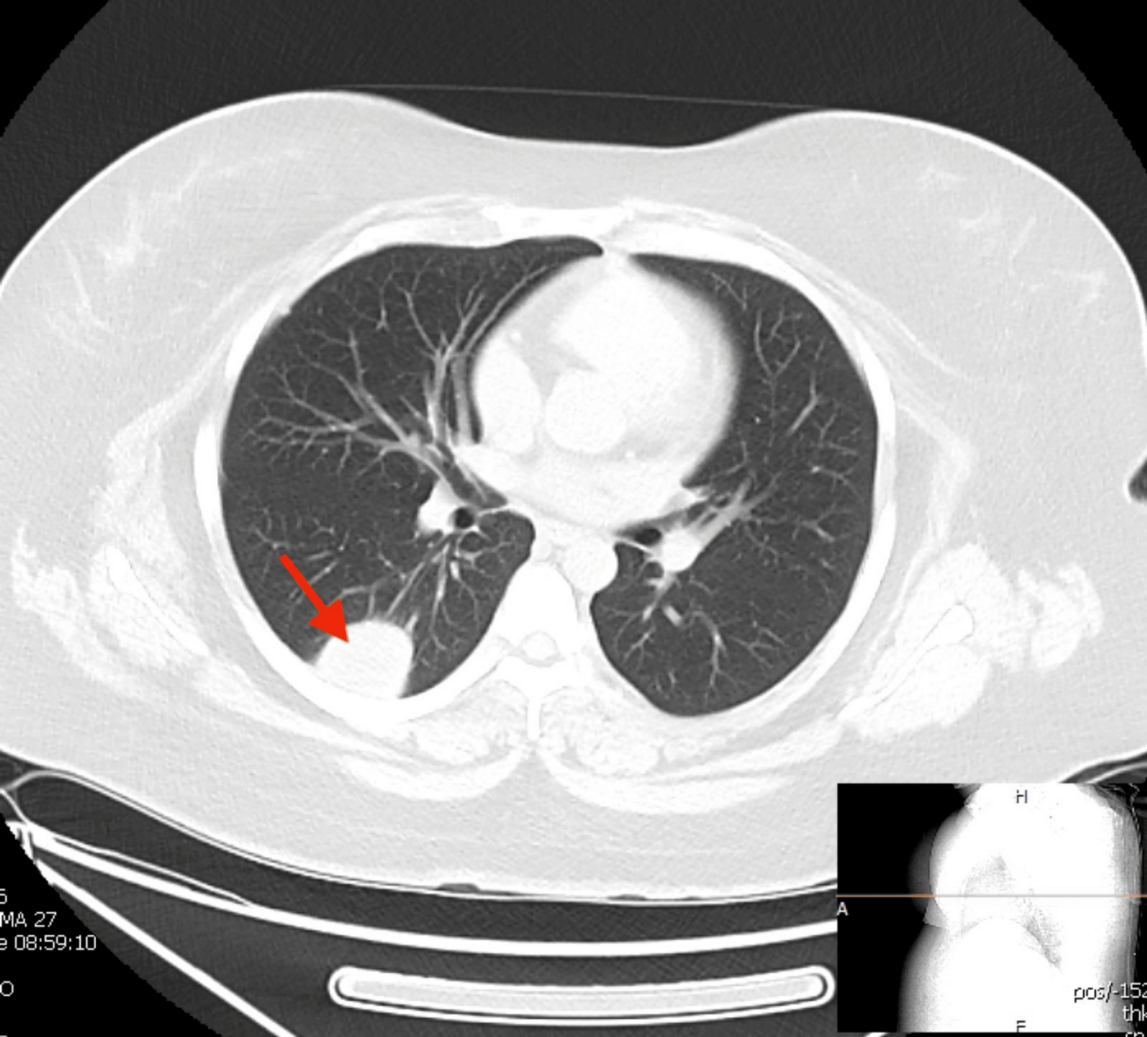
Computed tomography scan of the chest showing a pulmonary nodule. Image authorized by the patient.

Her baseline chest X-ray was unremarkable. She started with adalimumab 40 mg every two weeks and prednisone 5 mg daily three months prior. She reported having a COVID-19 infection one week later, which resolved. A month later, she developed a nonproductive cough with intermittent shortness of breath. She completed a course of azithromycin with no resolution, for which she underwent computed tomography (CT) scan of the chest, which showed multiple pulmonary nodules with a slightly greater than 3 cm rounded opacity within the right lower lobe (Figure 4).

**Figure 5:**
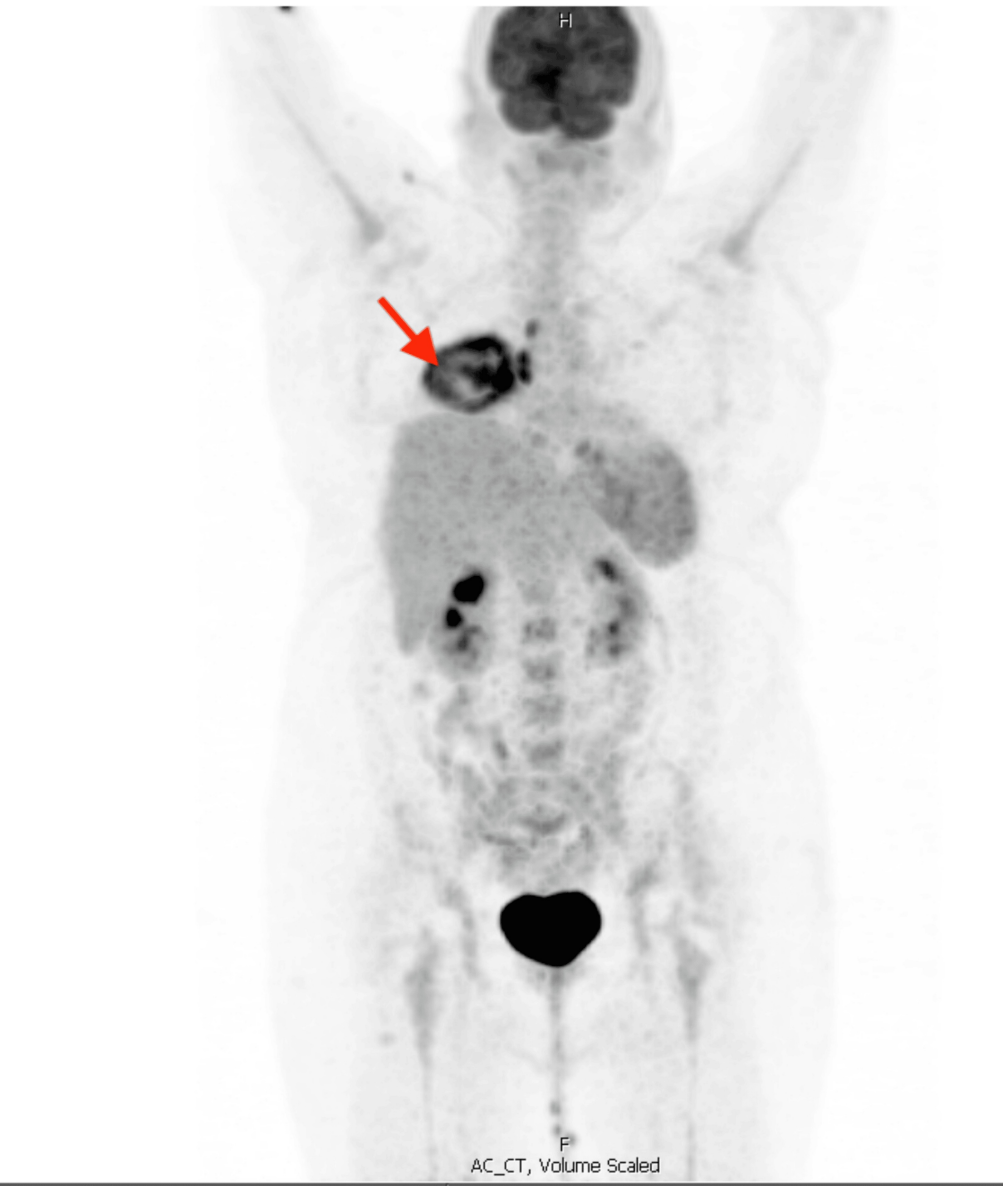
PET scan revealing an enlargement interval of the opacity of the right lower lobe nodule. Positron emission tomography (PET) scan image authorized by the patient.

A positron emission tomography (PET) scan was recommended (Figure 5). An enlargement interval of the opacity was demonstrated at 8.1 cm of the right lower lobe nodule, with at least three more minor tracer avid rounded opacities throughout the bilateral lung fields. Present also mildly enlarged bilateral submandibular lymph nodes and splenic involvement within one month.

Laboratory findings, as mentioned in (Table 1), are notable for leukocytosis, elevated liver function tests, and inflammatory markers. Autoimmune workup demonstrated antinuclear antibodies (ANA), anti-Sjögren's-syndrome-related antigen A (anti-SS-A) positive. High titer proteinase 3 (PR-3) antibody, c-ANCA, rheumatoid factor, and cyclic citrullinated protein. Negative anti-SS-B, Smith, ribonucleoprotein (RNP), myeloperoxidase antibodies, cryoglobulins, and anti-histone antibodies. Complement levels were normal. Urinalysis was abnormal; urine protein creatinine ratio was unremarkable. HIV, Nontreponemal venereal disease research laboratory (VDRL), Quantiferon TB gold plus, Hepatitis B, and Hepatitis C were non-reactive. The respiratory viral panel showed no pathogen detected. Skin biopsy showed leukocytoclastic vasculitis with direct immunofluorescence (DIF) studies for immunoglobulin (Ig)G, IgA, IgM, complement component 3 (C3), and fibrinogen, showing a non-specific/ non-diagnostic staining pattern. Transthoracic echocardiography was unremarkable. Bronchoscopy with lymph node biopsy demonstrated few lymphoid aggregates, histiocytes, and benign-appearing bronchial cells. The right lower lobe mass fine needle biopsy and flow cytometry showed lymphohistiocytic aggregates and reactive bronchial cells with no evidence of fungal infection or malignancy. 

**Table 1:** Laboratory values of the patient with reference values included. IgM: Immunoglobulin M; ANA: Antinuclear antibodies; C-ANCA: Cytoplasmic anti-neutrophil cytoplasmic autoantibody; P-ANCA: Perinuclear anti-neutrophil cytoplasmic antibodies: CCP: Cyclic citrullinated peptide; Anti-SS-A:anti–Sjögren's-syndrome-related antigen A; Anti-SS-B: anti–Sjögren's-syndrome-related antigen B.

Laboratory	Value	Reference range
Leukocytes	15.9	4.5-11 × 10^3^/cm^3^
Hemoglobin	13.8	12.0-16.0 g/dl
Platelets	469.000	140-440 × 10^3^/uL
Blood urea nitrogen	12	6-22 mg/dl
Creatinine	0.66	0.51-0.95 mg/dl
Aspartate aminotransferase	76	14-33 IU/l
Alanine transaminase	101	10-42 IU/l
Albumin	2.99	3.8-4.9 g/dl
Erythrocyte sedimentation rate	103	< OR = 20 mm/h
C-Reactive protein	>10	< 8.0 mg/l
Urinalysis	Protein 30. RBC 6	Negative mg/dl. Negative 0-5 HPF
Urine protein/creatinine ratio	93	24-184 mg/g creat
Serum Beta-Human chorionic gonadotropin	<5 mIU/mL	Negative: < 5 mIU/ml. Positive: > 5 mIU/ml
HIV	Non-reactive	Negative: Non-reactive. Positive: Reactive
Syphilis	Non-reactive	Negative: Non-reactive. Positive: Reactive
Hepatitis B	Non-reactive	Negative: Non-reactive. Positive: Reactive
Hepatitis C	Non-reactive	Negative: Non-reactive. Positive: Reactive
Ebstein Barr virus IgM	Negative	Negative: No infection. Positive: Acute infection
QuantiFERON- Tb Gold Plus	Negative	Negative
ANA titer	1:80	Positive
Atypical pANCA	<1:20	Negative < 1:20 titer
C-ANCA	1:320	Negative < 1:20 titer
P-ANCA	<1:20	Negative < 1:20 titer
Rheumatoid factor	>650	Negative <14 IU/ ml
CCP IgG antibodies	>250	Negative: <20. Strong positive >59
Proteinase-3 antibody	67.0	No antibody detected: < 1.0 AI. Antibody detected: > or = 1.0 AI
Myeloperoxidase antibody	<1.0	No antibody detected: < 1.0 AI. Antibody detected: > or = 1.0 AI
Complement C3	191	83-193 mg/dl
Complement C4	45	15-57 mg/dl
Cryoglobulin, qualitative	Negative	It may consist of cryoglobulins, fibrins, complement, or a mixture.
Ribonucleoproteins antibodies	<0.2	Negative: 0.0-0.9 AI
Smith antibodies	<0.2	Negative: 0.0-0.9 AI
Sjogren´s Anti-SS-A	4.9	Negative: 0.0-0.9 AI
Sjogren´s Anti-SS-B	<0.2	Negative: 0.0-0.9 AI
Anti-histone antibodies	0.7	Negative: <1.0 Units. Strong positive: >2.5

The diagnosis was reached based on the purpuric eruption, leukocytoclastic vasculitis histological findings, high titers C-ANCA/ PR3, and after excluding alternative diagnoses for the lung mass. A Birmingham Vasculitis Activity Score Version 3, a proven measure to assess the activity disease in patients with many different forms of vasculitis, was calculated with an initial score of 3 points and a new/worse score of 17. These scores indicated severe active AAV requiring aggressive vasculitis treatment. She was started on intravenous steroids and rituximab dosing of 375 mg/m^2^ every week for four doses, along with avacopan for steroid tapering. Untreated AAVs have a poor prognosis; nowadays, this has improved due to early identification and prompt treatment. Her outpatient follow-up showed no new skin lesions, stable respiratory symptoms, normal renal function, and hematuria resolution.

## Discussion

The overlap between GPA and RA in the context of TNF-inhibitor therapy presents a diagnostic and therapeutic challenge. This case underscores the need for vigilance in patients with RA who develop new symptoms suggestive of systemic vasculitis, especially when on TNF inhibitors like adalimumab [6]. The development of GPA in this patient, evidenced by clinical features such as nonproductive cough, pulmonary nodules, and palpable purpura, alongside laboratory findings including positive high titer c-ANCA and PR3 antibodies, points towards a complex interplay between RA and GPA or a potential drug-induced etiology. Several documented cases suggest that a COVID-19 infection can trigger ANCA-associated vasculitis (AAV3) [7]. A systematic review and case reports have indicated that SARS-CoV-2 infection might trigger AAV in genetically susceptible individuals [8].

The temporal association with TNF inhibitor therapy is particularly notable. TNF inhibitors are known to be effective in managing RA, but they can paradoxically induce autoimmune conditions, including vasculitis. In this patient, the onset of GPA symptoms following the initiation of Humira and the subsequent biosimilar suggests a possible drug-induced mechanism. This aligns with existing reports of TNF inhibitor-induced vasculitis, which often resolves upon discontinuation of the offending agent [9]. A comprehensive diagnostic workup is essential to exclude other potential causes of vasculitis, such as infections, malignancies, and other autoimmune diseases. In this case, the diagnostic process included a broad spectrum of tests, including CBC, Complete Metabolic Panel (CMP), acute phase reactants, autoimmune panels, and imaging studies, excluding current infection, exploring the differential diagnosis of small to medium vessel vasculitis [10]. Tissue sampling aided in confirming diagnosis and excluding other etiologies, which helped confirm GPA diagnosis. The negative infectious panel, normal complement levels, and absence of cryoglobulins and anti-histone antibodies further supported the diagnosis.

Treatment decisions in such overlap syndromes must be tailored to the individual patient, balancing the management of RA with the need to control GPA [9]. The use of rituximab, an alternative to TNF inhibitors, in conjunction with IV steroids, was chosen to induce remission for AAV. Rituximab is also indicated for RA, thus may help patients with overlapping conditions [7].

## Conclusions

We report a case of GPA overlaps with RA/developing GPA during Adalimumab therapy for RA. This case highlights the importance of considering drug-induced vasculitis in patients with RA presenting with new systemic symptoms. We recommend high suspicion for these entities in the setting of a long-standing history of RA with new onset of systemic symptoms, new clinical evidence of glomerulonephritis, upper or lower respiratory tract involvement, purpura or multiple mononeuropathy for a prompt recognition, comprehensive evaluation, diagnosis, multidisciplinary specialty involvement, initiation of therapy, and appropriate therapeutic adjustments are crucial for complex cases. This case adds to the limited but growing evidence of the rare coexistence of RA and GPA.
